# Mortality in neonates with giant omphalocele subjected to a surgical technique in Barranquilla, Colombia from 1994 to 2019

**DOI:** 10.1038/s41598-020-78991-y

**Published:** 2021-01-11

**Authors:** Alexander Barrios-Sanjuanelo, Cristóbal Abelló-Munarriz, Jaiberth Antonio Cardona-Arias

**Affiliations:** 1 Epidemiology, University of Antioquia, Medellin, Colombia; 2 North University (Universidad del Norte-Uninorte), Barranquilla, Colombia; 3 Metropolitan University (Universidad Metropolitana-UM), Barranquilla, Colombia; 4 Minimally Invasive, High-Complexity Pediatric Surgery Group, CMI International Pediatrics Clinic (Clínica CMI Pediátrica International), Barranquilla, Colombia; 5 School of Microbiology, University of Antioquia, Medellin, Colombia

**Keywords:** Gastrointestinal system, Gastrointestinal diseases, Paediatrics, Therapeutics

## Abstract

No studies of the efficacy and safety of surgical techniques for the primary closure of giant omphalocele have been performed in Colombia. To determine the mortality rate and factors associated with mortality in neonates with giant omphalocele subjected to the surgical technique of early closure with a surgical silo described by Abello in Barranquilla, Colombia from 1994 to 2019. Retrospective cohort study of 30 neonates diagnosed with giant omphalocele and subjected to early closure of the defect. Medical history data were collected, information bias was controlled for, and descriptive statistical analysis was performed using Fisher’s exact test and the Mann–Whitney U test in SPSS 25.0. Of the patients in the cohort, 36.7% presented technique-related complications, 56.7% developed sepsis, 23.3% had low birth weight, 26.7% were preterm births, 43.3% had other malformations, 26.7% had congenital heart defects, and 13.3% presented pulmonary hypertension. The mean hospital stay was 26 days. The mortality rate was 16.7%; it was significantly higher among patients with other malformations, congenital heart defects, pentalogy of Cantrell and pulmonary hypertension. The Abello technique for the treatment of giant omphalocele showed a high neonatal survival rate and a low rate of procedure-related complications. The main factors associated with the death of neonatal patients were the presence of other malformations, congenital heart defect, pentalogy of Cantrell and pulmonary hypertension.

## Introduction

Congenital abdominal wall defects are the most common surgical problem among fetuses and neonates^1^. Omphalocele is an abdominal wall midline defect of variable size with herniated viscera; it may include the intestine, part of the liver and other organs depending on the size and location of the defect^2,3^. Giant omphalocele is an extreme version of this defect, although there is currently no consensus on its definition^4^. Surgeons usually define giant omphalocele as a defect 5 cm or larger in diameter; 37–67% of cases are associated with additional congenital anomalies such as Beckwith–Wiedemann syndrome and pentalogy of Cantrell^5^.

In Japan, the prevalence of omphalocele from 1997 to 2006 was one case per 2500 births. The incidence in live births is 1 in 4000 due to a very high rate of termination of pregnancy (30–52%) resulting from the presence of associated abnormalities and spontaneous abortion^2,6^. In the International Clearing house for Birth Defects Surveillance and Research Annual Report 2012, the Bogotá Congenital Malformations Surveillance Program reported a prevalence of omphalocele in Colombia of 3.03 cases per 10,000 births in 2010^7^. For 2016, according to the monthly newsletter reports of the Bogotá Congenital Malformations Surveillance Program, a prevalence of 1.85 cases of omphalocele per 10,000 births was calculated^8^. Its occurrence has been associated with gender, mother’s age, tobacco smoking and alcohol consumption^4^.

Morbidity and mortality rates are directly correlated with the presence and severity of anatomical and chromosomal abnormalities, and ranged from 13 to 25% among infants with small omphaloceles. These rates are even higher among children with giant omphalocele due to the larger size of the abdominal wall defect, viscera-abdominal disproportion and possible associated anomalies such as Beckwith-Wiedemann syndrome; pulmonary hypoplasia; congenital heart defects; trisomy 13, 15, 16 and 18; and pentalogy of Cantrell^5^.

Surgical management of omphalocele varies with defect size and type, newborn size and associated conditions. Treatment of this condition aims to reduce herniated abdominal viscera and to close the fascia and skin to create a solid abdominal wall without creating excessive intra-abdominal pressure^2,3^. The techniques available including Gross technique in which the defect is covered with skin flaps preserving the amnion, Schuster technique which consists of using a surgical silo attached to the fascia with nonabsorbable sutures, and other assequential ligation of the sac, use of tissue expanders and nonoperative escharotic therapy^9^. When these techniques are used, high mortality and complications such as surgical wound or fascial infection, dehiscence, enterocutaneous fistula, sepsis and general difficulties closing the abdomen, with subsequent death, have been reported^10^.

The Abello technique presents several advantages as a less invasive approach that does not require the use of sutures. In this technique, a pressure vector is generated that reduces the content until simulating definitive closure, thereby making it possible to handle giant omphaloceles with shorter defect reduction times and as an early surgical repair technique that improves the prognosis and increases the survival rate and quality of life of neonates with giant omphalocele^11^.

The factors associated with mortality in this disorder or with prognosis after intervention include the presence of associated abnormalities, extracorporeal liver, preterm birth, rupture of the sac, respiratory insufficiency primarily due to increased abdominal pressure at the time of surgical repair and pulmonary hypertension, as well as postsurgical complications such as pneumonia, sepsis and bowel necrosis and intestinal occlusion secondary to the formation of intestinal bands and intestinal malrotation, among others^1,4,12–15^.

Few research studies on giant omphalocele are available, and most are case series, with limited reporting of treatment efficacy or treatment failures^1,5^. In a study conducted in the United States that included more than 2000 cases over a period of 10 years, the total mortality rate was 28.7%, with a hazard ratio (HR) of 7.7 among neonates with chromosomal abnormalities and an HR of 7.5 for cases with low birth weight^4^. This finding suggests that defect size and congenital defects are not the only factors associated with mortality.

The objective of this study was to determine the mortality rate and the factors associated with it in neonates with giant omphalocele subjected to a surgical technique described by Abello in Barranquilla, Colombia from 1994 to 2019.

## Methods

### Study type

Observational retrospective cohort study.

### Study subjects

Thirty neonates diagnosed with giant omphalocele (abdominal wall defect > 5 cm and/or liver content) who were operated on using the Abello technique in Barranquilla from 1994 to 2019. Neonates with giant omphalocele who were subjected to a surgical technique other than surgical silo were excluded from the study.

### Abello technique description

The Abello technique aims to evaluate the newborn at birth and to perform in three phases. In the silo or phase-one management, two 20 × 20 cm DuoDERM CGF hydrocolloid dressings are used to prepare the surgical silo, gradually reducing and pushing the visceral content into the abdomen until it lies flat with the liver inside the abdominal cavity; in the second phase, amnion inversion and amnion and skin closure are performed to bridge the skin edges, thereby simulating definitive closure for phase three, which consists of definitive closure of the defect in the operating room. This technique is based on biomechanical principles; that is, a surgical silo provides the same traction effect as sutured silos by spreading the force over an area and not over a suture line, distributing the force over a higher number of points without requiring sutures and thereby noninvasively achieving the same effect while ensuring amnion preservation and inversion.

**Figure 1:**
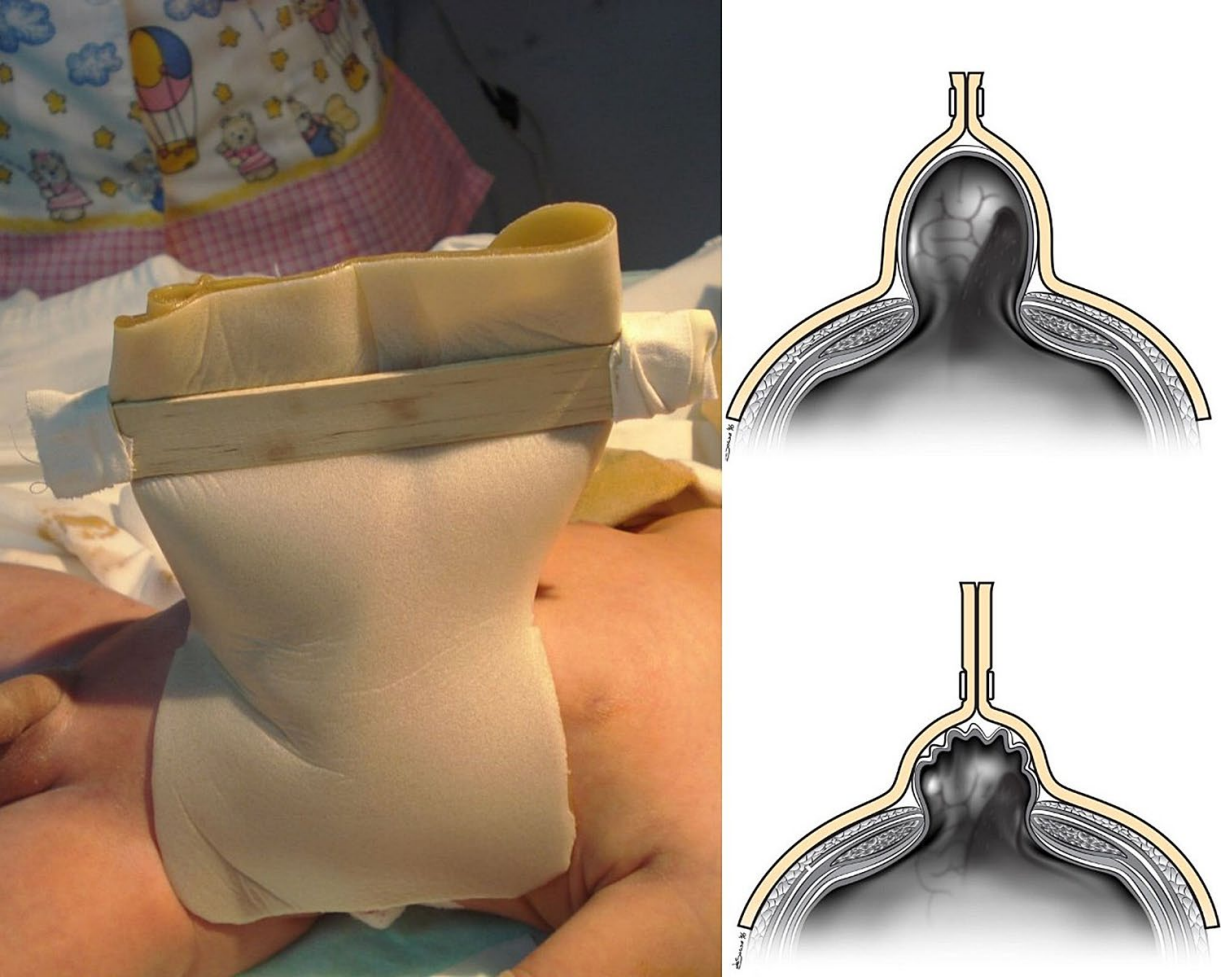
Phase 1 or preparing of the silo and starting the gradual reduction.

Description of the phases:

The first phase (Fig. 1) consists of preparing the silo and starting the gradual reduction, day by day, according to the tolerance to silo compression, using a pair of tongue depressors as clamps in the apex of the silo and maintaining a maximum pressure of 20 mmHg. The urine flow or output is monitored; in case of oliguria in the following 4 h, the pressure should be lowered, making the necessary adjustments to the respirator according to the cardiorespiratory effects generated by the increase in intra-abdominal pressure; inspiratory pressure, FiO_2_ and respiratory rate can be adjusted to maintain acid–base balance. These changes are usually necessary due to the tendency towards respiratory acidosis, hypercapnia and hypoxemia.

**Figure 2:**
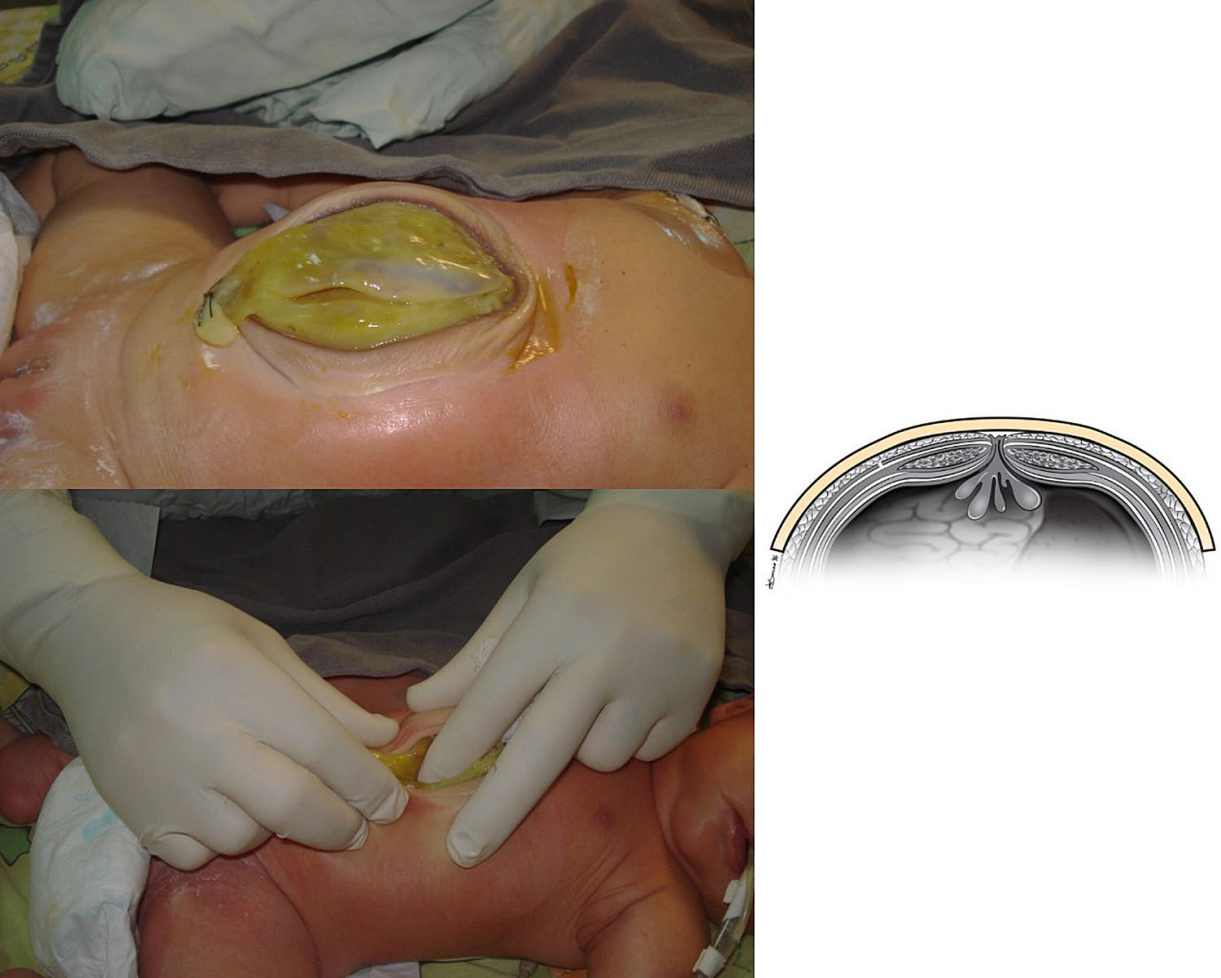
Phase 2 or skin closure with gradual amnion inversion.

The second phase (Fig. 2) begins after completion of the reduction in the visceral content of the sac. This phase consists of performing amnion and skin closure with gradual amnion inversion until the amnion and skin edges are bridged, simulating definitive closure and checking the patient’s tolerance.

**Figure 3:**
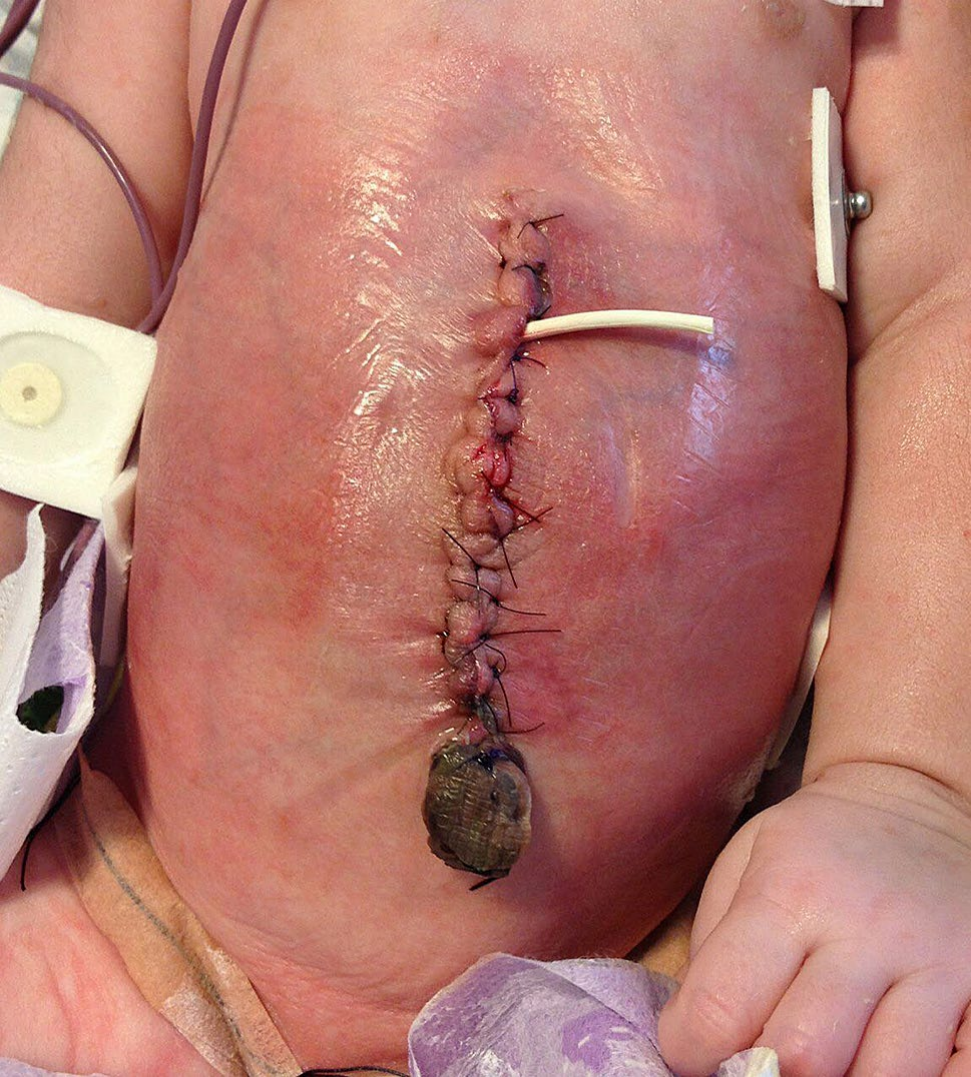
Phase 2 or definitive closure of the defect.

The third phase (Fig. 3) corresponds to definitive closure of the defect in the operating room by amnion resection, review of the abdominal cavity for the possibility of abnormal intestinal rotation or other anomalies and layered suturing of fascia and skin, with umbilicoplasty in virgin and intact skin for improved esthetic and functional results without disregarding the possibility of management with second-intention healing (9).

Since 1994, no changes have been made to the described procedure.

### Data collection

A secondary information source (medical records of the patients) consisting of the study subjects’ clinical histories was used in this study. The variables survival (follow-up period until hospital discharge or death of the participant), gestational age, newborn gender, birth weight, associated malformations, congenital heart defects, pentalogy of Cantrell, pulmonary hypertension (by echocardiography), condition of the sac, duration of hospital stay, time of silo reduction, time of inversion and reduction in the sac, time of abdominal wall closure without tension, abdominoplasty, component separation, surgical and nonsurgical complications, botulinum toxin and sepsis were extracted from this source. Complications were classified into two groups: (a) related to the technique, correspond to complications secondary to the increase in intra-abdominal pressure to correct the defect (for example, ascites, lower limb edema, hydrocele, oliguria and necrosis); (b) complications not related to the technique which are associated with abdominal wall defect (for example congenital heart disease) or diseases acquired during the stay in the neonatal unit (sepsis, pneumonia, atelectasis, pulmonary hypertension).

Double data extraction from medical records, with contingency tables or logical data analysis, was performed to control for biases.

### Statistical analysis

The population was described using central tendency and frequency measures to identify factors associated with mortality in the study group. Fisher’s exact test was used to compare dichotomous variables, and the Mann–Whitney U test was used to compare quantitative variables because the assumption of normality was not met, as shown by the Shapiro–Wilk test. The tests were performed in SPSS 25.0 using a significance level of 0.05.

### Ethical considerations

The guidelines set forth in the Declaration of Helsinki and in Resolution 8430 of the Ministry of Health of Colombia (1993) were followed in this study. The project was evaluated by the research ethics committee of CES University. At the time of performing the surgical procedure, the parent or legal guardian of minor signed an informed consent that endorses the use of clinical data for academic and research purposes.

## Results

Most affected newborns were girls (56.7%); 23.3% had low birth weight, 26.7% were preterm births, and a significant proportion presented other malformations and diseases in addition to giant omphalocele (Table 1).

**Table 1:** Clinical characteristics of the study subjects.

Variables and their factors or levels		n	%
Birth weight	Low (< 2500 g)	7	23.3
	Adequate (≥ 2500 g)	23	76.7
Gestational age at birth	Preterm	8	26.7
	Term	22	73.3
Other characteristics of the newborn	Malformations	13	43.3
	Congenital heart defects	8	26.7
	Pulmonary hypertension	4	13.3
	Pentalogy of cantrell	2	6.7

	Mean ± SD	Median (IQR)	Range
Weight in kilograms	2.85 ± 0.59	2.95 (0.26–3.2)	1.13–3.9
Weeks of gestation	37.2 ± 2.4	38 (36–39)	28–40

*SD* standard deviation, *IQR* interquartile range.

Ninety percent of the newborns in the study had an intact omphalocele sac at birth; 70% of the newborns were hospitalized for 15–30 days; more than 60% showed silo reduction, sac inversion and abdominal wall closure without tension within 1 week; 56.7% had sepsis; and 36.7% had other complications related to the technique (Table 2).

**Table 2:** Clinical and surgical characteristics of the study population.

Variables and their factors or levels		n	%
Condition of the omphalocele sac	Intact	27	90.0
	Ruptured	3	10.0
Hospitalization days	< 15 days	2	6.7
	15–30 days	21	70.0
	> 30 days	7	23.3
Silo reduction	Yes	24	80.0
	No	6	20.0
Time (days)	Silo reduction < 7 days	24	80.0
	Sac inversion < 5 days	20	66.7
	Complete sac reduction < 10 days	20	66.7
	Abdominal wall closure without tension < 10 days	20	66.7
	Abdominoplasty < 15 days	22	73.3
Other characteristics	Mesh overlay	2	6.7
	Mesh reinforcement	5	16.7
	Use of component separation technique	6	20.0
	Use of botulin toxin	3	10.0
Complications	Related to the technique	11	36.7
	Unrelated to the technique	18	60.0
	Sepsis	17	56.7

Time (in days)	Mean ± SD	Median (IQR)	Range
Hospital stay	25.9 ± 12.4	23 (18–28)	12–61
Silo reduction	5.4 ± 3.4	5 (4–7)	0–13
Sac inversion	5.2 ± 1.8	5 (4–6)	2–9
Complete sac reduction	10.6 ± 4.3	9 (8–12)	5–22
Abdominal wall closure without tension	10.8 ± 3.8	10 (8–12)	5–22
Final abdominoplasty	13.6 ± 5.6	12 (10–16)	6–35

*SD* standard deviation, *IQR* interquartile range.

A mortality rate of 16.7% (n = 5) was found among the study subjects. Mortality was significantly associated with the presence of other malformations, congenital heart defects, pentalogy of Cantrell and pulmonary hypertension. No significant association was found between mortality and the condition of the omphalocele sac, the presence of sepsis or other complications unrelated to the technique, or silo reduction, sac reduction and abdominal wall closure and abdominoplasty times. The other study variables failed to show sufficient statistical power to justify a comparison of newborns who died with those who survived (Table 3).

**Table 3:** Factors potentially related to the mortality of the study subjects.

Factors associated with mortality		n	Death % (n)	p Fisher	Power %
Malformations	Yes	13	38.5 (5)	0.009	99
	No	17	0.0 (0)		
Congenital heart defects	Yes	8	50.0 (4)	0.011	96
	No	22	4.5 (1)		
Pentalogy of Cantrell	Yes	2	100.0 (2)	0.023	93
	No	28	10.7 (3)		
Pulmonary hypertension	Yes	4	75.0 (3)	0.009	96
	No	26	7.7 (2)		
**Nonassociated factors**					
Presence of sepsis	Yes	17	29.4 (5)	0.052	97
	No	13	0.0 (0)		
Complications unrelated to the technique	Yes	18	27.8 (5)	0.066	96
	No	12	0.0 (0)		
Condition of the omphalocele sac	Intact	27	18.5 (5)	1.000	91
	Ruptured	3	0.0 (0)		
Silo reduction time	< 7 days	24	20.8 (5)	0.553	92
	> 7 days	6	0.0 (0)		
Total time for complete sac reduction	< 10 days	20	25.0 (5)	0.140	95
	> 10 days	10	0.0 (0)		
Total time for abdominal wall closure without tension	< 10 days	20	25.0 (5)	0.140	95
	> 10 days	10	0.0 (0)		
Abdominoplasty	< 15 days	22	22.7 (5)	0.287	93
	> 15 days	8	0.0 (0)		
**Comparisons with low statistical power**					
NB gender	Male	13	23.1 (3)	0.628	27
	Female	17	11.8 (2)		
Mesh overlay	Yes	2	50.0 (1)	0.310	23
	No	28	14.3 (4)		
Silo reduction	Yes	24	16.7 (4)	1.000	5
	No	6	16.7 (1)		
Mesh reinforcement	Yes	5	20.0 (1)	1.000	7
	No	25	16.0 (4)		
Use of the component separation technique	Yes	6	16.7 (1)	1.000	5
	No	24	16.7 (4)		
Use of botulin toxin	Yes	3	0.0 (0)	1.000	50
	No	27	18.5 (5)		
Complications related to the technique	Yes	11	18.2 (2)	1.000	7
	No	19	15.8 (3)		

Table 4 presents the analysis of mortality according to the quantitative study variables; none of these variables showed significant differences.

**Table 4:** Mortality analysis of the study subjects according to their quantitative clinical and surgical characteristics.

Variable	Death	Survival	p
	X ± DE	X ± DE	
Weight (kg)	2.89 ± 0.43	2.84 ± 0.63	0.860^a^
Silo reduction time (days)	3.2 ± 2.0	5.8 ± 3.5	0.126^a^
Sac inversion time (days)	5.0 ± 2.1	5.2 ± 1.8	0.790^a^

	Me (IQR)	Me (IQR)	
Weeks of gestation	38 (37–39)	38 (36–39)	0.589^b^
Days of hospital stay	23 (19–23)	24 (18–31)	0.481^b^
Total time for complete sac reduction (days)	8 (8–8)	10 (8–14)	0.152^b^
Total time for abdominal wall closure without tension (days of life)	9 (8–9)	10 (8–14)	0.275^b^
Final abdominoplasty (days of life)	10 (10–12)	12 (10–17)	0.251^b^

*X* mean, *SD* standard deviation, *Me* median, *IQR* Interquartile range.

^a^Student’s t test for independent samples with equal variances.

^b^Mann–Whitney U test.

In 100% of cases in which the newborn died, he or she showed malformations, sepsis, intact omphalocele sac and no record of complications related to the technique. Table 5 presents other characteristics of the cases in which death occurred.

**Table 5:** Summary table of the clinical characteristics of the infants who died.

	Case 1	Case 2	Case 3	Case 4	Case 5
Birth weight (kg)	2.300	2.950	2.810	3.500	2.900
Gender	Male	Male	Female	Female	Male
Gestational age (weeks)	39	39	38	37	37
Congenital heart defect	No	Yes	Yes	Yes	Yes
Pentalogy of Cantrell	No	No	No	Yes	Yes
Pulmonary hypertension	No	Yes	Yes	Yes	No
Silo reduction	No	Yes	Yes	Yes	Yes
Mesh overlay	No	No	No	Yes	No
Component separation	No	No	No	No	Yes
Botulin toxin	No	No	No	Yes	No
Other complications	Yes	Yes	No	No	No
**Time in days**					
Hospitalization	16	19	23	23	24
Sac inversion	8	3	5	6	3
Complete sac reduction	8	8	8	9	8
Abdominal wall closure	8	10	9	9	8
Abdominoplasty	10	12	10	12	10

## Discussion

This is the first study of giant omphalocele conducted in Colombia in which treatment efficacy (number of surviving patients) and safety (complications related to the technique) data for a new technique based on early defect closure in the neonatal stage, as well as the factors associated with the mortality of patients who were subjected to the procedure, were evaluated.

This study showed that mortality among newborns with giant omphalocele subjected to the Abello surgical technique in the city of Barranquilla was 16.7% (83.3% survival). Mortality was associated with the presence of other malformations, congenital heart defects, pentalogy of Cantrell and pulmonary hypertension. The studies of Mitanchez et al*.*^16^ and Roux et al*.*^17^, which employed sample sizes similar to the sample size in this study, reported similar mortality and concluded that anomalies associated with the condition can be a decisive factor in survival, particularly in the case of serious heart defects, as described in this Colombian population. It is important to clarify that similar studies are not known in Colombia, so there is no good local comparator, even in the same institution of the patients of this study, there are no data available about subjects undergoing other procedures surgical.

Other studies have reported survival rates lower than 20% or 50% in cases diagnosed prenatally (including termination), whereas studies examining postnatal survival also reported a close association with the presence and severity of anatomical and chromosomal abnormalities^1^. In the absence of structural anomalies or chromosomal abnormalities, most infants with smaller omphaloceles have a 1-year survival rate of 92% with no long-term problems, according to the results of a study conducted in the United Kingdom, although the 1-year survival rate decreased to 27% in infants with chromosomal abnormalities^4^.

This study faced difficulties in categorizing the type of congenital heart defects present in the infants due to limitations in echocardiographic outcomes. Despite these difficulties, the prevalence of congenital heart defects in this study was lower than that reported in other studies such as the study of Gibbin et al*.*^18^, in which ventricular and atrial septal defects were the most frequent. These results highlight the importance of performing both prenatal and postnatal echocardiography in these cases.

After congenital heart defects, pentalogy of Cantrell was another major cause of death among newborns with giant omphaloceles. Some of these cases were not reported because this condition is cause for termination of pregnancy in some countries^19^. Some case reports show higher 1-year survival rates when the patients are subjected to late defect closure^20^. In this study, the two reported cases died, albeit for causes unrelated to the surgical procedure. Consequently, early defect closure has recently been considered a factor that increases the risk of death^21^.

As in this study, other authors have found an association between mortality and pulmonary hypertension, a condition that is observed in more than a third of patients with giant omphaloceles^14^. Abnormal pulmonary vascular tone is frequently implicated in pulmonary hypoplasia and represents a significant limitation to survival and long-term functional outcomes. Panitch states that newborns with giant omphalocele are at risk for pulmonary arterial hypertension, which increases the risk for mortality in the neonatal period^22^. For Baerg et al*.*, pulmonary hypertension is a predictor of mortality when this condition occurs between the second and seventh days of life^23^. The above findings demonstrate that pulmonary hypertension is a significant and underestimated complication and highlight the importance of its early (after the second day of life) and periodic monitoring by echocardiography among patients with giant omphalocele^14,24^.

In contrast to the main findings of the present study, Amulya K. Saxena, who conducted a systematic review of 23 articles covering 396 cases to determine mortality predictors in giant omphalocele, found a significant association between death and gestational age, birth weight, the presence of eviscerated organs and associated anomalies^25^. This finding highlights the need to perform studies that are specific to each country or region because only four variables were significant in Barranquilla–Colombia (malformations, congenital heart defects, pentalogy of Cantrell and pulmonary hypertension).

The present study found no association between mortality and the condition of the omphalocele sac, the presence of sepsis or other complications unrelated to the technique and silo reduction time, sac reduction time, abdominal wall closure time and abdominoplasty time. However, according to the available scientific literature, other variables that have been related to mortality include defect size, the presence of extracorporeal liver, prematurity and ruptured sac^4^, respiratory failure mainly due to increased abdominal pressure at the time of surgical repair or intrauterine lung growth failure, surgical complications (pneumonia and sepsis), intestinal necrosis and increased intra-abdominal pressure at the time of defect closure, such as decreased lung distensibility or alterations of the urinary system secondary to poor renal perfusion and intestinal occlusion events secondary to the formation of intestinal bands^15^.

Comparison of the findings of this study with findings in other populations is not easy because newborns with giant omphalocele are less likely to undergo treatment with primary closure and are treated with late closure in most cases due to the significant impact of the defect size^26^. Accordingly, the study by the Bauman group, which was based on a systematic review of 14 studies comprising 350 patients, describes as study variables the available surgical techniques and mortality and concludes that despite advances in medical and surgical treatments, giant omphalocele is still associated with a high mortality rate and numerous morbidities. The authors of that study recommend using late nonsurgical treatment as a first-line treatment for newborns with giant omphalocele^5^.

In addition to the above, a series of long-term medical problems associated with giant omphalocele, such as gastroesophageal reflux, pulmonary insufficiency, recurrent lung infections or asthma and feeding difficulties with growth failure, must also be monitored^6^. These problems are even more serious considering the results of a survey conducted by a group of surgeons who concluded that, for 30 years, no fully accepted technique has been available for the treatment of giant omphalocele^27^. These problems, together with the risk of death of newborns with giant omphalocele and the multiple factors associated with survival, highlight the need to continue research on new therapeutic options that can overcome the limitations of currently available treatments, such as the Abello technique analyzed in this patient cohort.

Among the limitations of this study, some problems of the Colombian healthcare system in following patients stand out. These problems occur because the healthcare services are passive (only those who visit medical centers are treated, and there is no proactive search for pregnant women to ensure that they begin prenatal care) and because multiple geographical and economic barriers to medical care access persist, particularly in some medical specialties. The diversity of the diagnostic criteria for giant omphalocele should also be considered; these criteria may differ with respect to sac diameter, abdominal wall defect diameter, inability to perform primary closure of the abdominal wall defect, tissue defect larger than 5 cm, liver and visceral hernia and disproportion of volume between the abdominal viscera and the abdominal cavity. Further efforts must be made to reach a consensus on its definition^5^. Statistically, although the study included patients over a period longer than 20 years, the number of cases was low, which precluded obtaining associations with good statistical power for a significant number of clinical and surgical variables.

The results of this study show that the use of the Abello technique for the treatment of giant omphalocele results in a high newborn survival rate and a low rate of complications related to the procedure; the main factors associated with newborn mortality were the presence of other malformations, congenital heart defects, pentalogy of Cantrell and pulmonary hypertension. These results are highly significant considering the small number of therapeutic options for this malformation, its crucial role for deciding the termination of pregnancy in severe cases, the difficulties in achieving primary closure and the limited number of studies on mortality in Latin America. These factors contribute to the overall importance of the evidence presented here for guiding measures towards improving survival or increasing the number of intervention options available for neonatal surgeons and neonatologists, families and other interested parties.
